# Preoperative Methods to Reduce Liver Volume in Bariatric Surgery: a Systematic Review

**DOI:** 10.1007/s11695-015-1769-5

**Published:** 2015-06-30

**Authors:** J. van Wissen, N. Bakker, H. J. Doodeman, E. P. Jansma, H. J. Bonjer, A. P. J. Houdijk

**Affiliations:** Department of Surgery, Medical Center Alkmaar, Wilhelminalaan 12, 1815 JD Alkmaar, The Netherlands; VU Medical Center, Amsterdam, The Netherlands; Trial Center Holland Health, Alkmaar, The Netherlands

**Keywords:** Bariatric surgery, Laparoscopic Roux-en-Y gastric bypass, Preoperative care, Preoperative diets, Liver, Liver volume

## Abstract

**Background:**

Patients qualified for gastric bypass surgery have an enlarged and fatty liver. An essential step in gastric bypass surgery is elevation of the left liver lobe to expose the gastroesophageal junction. An enlarged and fatty liver complicates the surgical procedure and increases the risk for laceration of the liver. The aim of our study was to evaluate methods to reduce liver volume in patients prior to gastric bypass surgery.

**Methods:**

A systematic literature search of multiple databases, including PubMed, EMBASE.com, and the Cochrane Library and a hand search of reference lists, was performed. We used the search terms morbid obesity and liver, including their synonyms and controlled terms. Inclusion criteria were as follows: patients with morbid obesity who qualified for bariatric surgery, the use of a preoperative treatment to reduce liver volume, and the use of imaging techniques before and after treatment.

**Results:**

In total, 281 patients in 11 different studies were included. Preoperative diets reduced liver size by an average of 14 %, alternative methods including nutritional supplements, reduced liver size between 20 and 43 %, and an intragastric balloon by 32 %.

**Conclusions:**

This review showed that nutritional supplements and intragastric balloon are more effective than low calorie diets in reducing liver volume prior to gastric bypass surgery. However, low calorie diet is the preferable method to reduce liver volume, considering the level of evidence and practical applicability. There is a need for well-designed randomized studies with sufficient power in order to confirm the effectiveness of preoperative methods to reduce liver volume.

## Introduction

Morbid obesity is a growing global problem with an increasing number of patients in need for bariatric surgery. In the past decade, the prevalence of morbid obesity in the USA has increased by 70 %, from 2.1 % in 2000 to 3.7 % in 2010 [1].

Laparoscopic Roux-en-Y gastric bypass (LRYGBP) is the gold standard in Europe and the USA and successfully reduces weight in patients with morbid obesity [2]. Patients qualified for LRYGBP often have an enlarged and fatty liver. The prevalence of steatosis and steatohepatitis in patients qualified for bariatric surgery varies between 52 and 90 % and between 33 and 89 %, respectively [3–9]. An enlarged and fatty liver complicates LRYGBP in two ways. First, the enlarged left liver lobe complicates the approach to the gastroesophageal junction. Second, the soft fatty liver is vulnerable and thereby increasing the risk of bleeding upon surgical manipulation. For these reasons, an enlarged and fatty liver is the most common reason for conversion from laparoscopic to open Roux-en-Y gastric bypass [10]. The overall conversion rate in LRYGBP is approximately 4 %, and an enlarged liver is responsible for approximately 50 % of the conversions [11].

Currently, a preoperative low calorie diet is recommended in patients qualified for LRYGBP in order to reduce liver volume and facilitate surgery [12–22]. The duration and content of the diet differ per country and per institution. There are also pharmacological and endoscopic measures to reduce liver volume that could be an alternative for preoperative diets.

The objective of this systematic review was to investigate the effectiveness of different preoperative methods to reduce liver volume in patients qualified for bariatric surgery.

## Methods

### Data Sources

We conducted a systematic literature search to identify studies relevant to the review. Initially broad search terms were used, including [MORBID OBESITY], [PREOPERATIVE CARE], [LIVER VOLUME], AND [BARIATRIC SURGERY], including their synonyms and controlled terms. The search was performed in multiple databases, including PubMed, EMBASE.com, and the Cochrane Library (via Wiley) from inception to November 6, 2013. The search strategy for PubMed included the following search terms: “Obesity, Morbid” [Mesh] OR “morbid obesity” [tiab] OR “morbidly obese” [tiab] OR “excess body weight” [tiab] AND “Liver” [Mesh] OR “liver” [tiab]. The search strategy for EMBASE included the following search terms: “morbid obesity”/exp OR “morbid obesity”:ti,ab OR “morbidly obese”:ti,ab OR “excess body weight”:ti,ab AND “liver”/exp OR liver:ti,ab. The search strategy for the Cochrane Library included the search terms “morbid obesity” AND “liver.” The electronic search was supplemented by a hand search of reference lists of the available relevant literature. The search was performed by one of the authors (EJ).

### Data Selection

Studies focusing on preoperative methods to reduce liver volume in patients qualified for bariatric surgery were included. The exact inclusion criteria were as follows: studies in patients undergoing bariatric surgery for treatment of morbid obesity, studies that investigated methods to reduce liver volume as pretreatment before bariatric surgery, and studies that used radiographic measurements of total liver volume or volume of left hepatic lobe.

### Statistical analysis

A meta-regression analysis on the studies focusing on the effect of preoperative LCD’s was performed. The separate effect of the amount of calories and the duration of the LCD on liver volume was analyzed, taking the number of patients in each study as a weighing factor. Statistical analyses were performed using SPSS (Statistical Package for the Social Sciences, version 20, 2009, Chicago, IL, USA).

## Results

### Study Characteristics

**Fig. 1:**
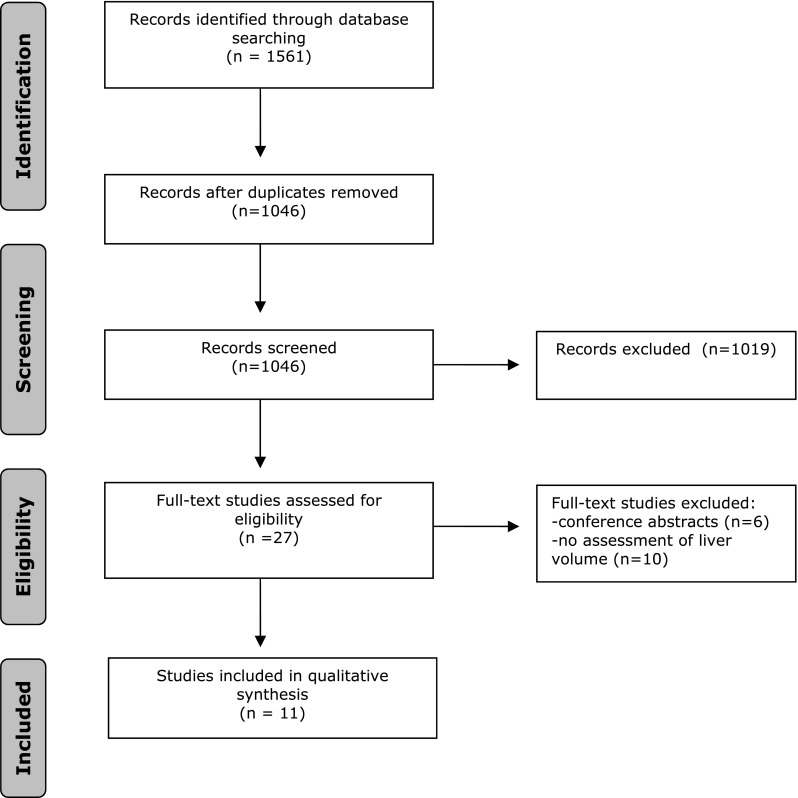
Flowchart of study inclusion

The initial search yielded a total of 1897 references. Two researchers (JW and NB) independently screened titles and abstracts for relevance. After removing duplicates of references that were selected from more than one database, 1310 references remained. From these, 1283 references were excluded because they were not relevant to the study. From the remaining 27 articles, full texts were screened for relevance. Finally, 11 studies were found eligible involving a total of 281 patients. The process of data selection is summarized in Fig. 1.

### Data Evaluation

In total, 9 prospective observational studies, 1 retrospective observational study, and 1 retrospective case-matched-control study were included. Seven studies (60 % of all patients) investigated the effect of a preoperative low calorie diet, 3 studies (29 % of all patients) focused on preoperative pharmacological measures, and in 1 study (11 % of all patients), the effect of a preoperative intragastric balloon on liver volume was evaluated. Overall, 59 % of the patients were women, 27 % were men, and in 14 %, gender was not described. The mean age of the studied patients varied from 34 to 48 years of age. The nine cohort studies that were included showed considerable heterogeneity in terms of patient population, sample sizes, intervention methods, and assessment of outcome. Only two studies had a controlled design. Edholm et al. [17] used a control group with the same gender and approximately the same sample size as the intervention group. The method of selection of the control group was not reported. Aberle et al. [12] used a retrospectively matched control group.

### Preoperative Low Calorie Diet

In total, seven studies [13, 15–18, 20, 22] investigated the effect of a preoperative low calorie diet on liver volume. The median duration of the preoperative diet was 6 weeks (range 2–12 weeks). The amount of calories ranged from a minimum of 456 kcal/day to a maximum of 1520 kcal/day. Body mass index (BMI) before preoperative diet ranged from 43 to 56 kg/m^2^. The median change in BMI after the low calorie diet was a reduction of 9.1 % (range 4.2–12.5 %). The median change in liver volume was a reduction of 15 % (range 5–20 %). The weighted average of change in liver volume was 14 %. Generally, longer duration of the diet showed more reduction in liver volume. However, the maximum percentage of change in liver volume did not correspond with the maximal duration of the diet or the minimum amount of calories per day. An overview of the effect of preoperative low calorie diets is shown in Table 1.

**Table 1:** Overview of preoperative low calorie diets

Year	Investigator	Study type	Patients (*n*)	Duration (weeks)	Calories (kcal/day)	BMI (% change)	Methods		Change in liver volume (%)	Change in liver volume per week (%)
							Liver or left lobe	Imaging technique		
2007	Benjaminov	Prospective	14	4	1520 ± 285	−5.1	Liver	CT	−8.2	−2.1
2006	Colles	Prospective	32	12	456–680	−10.6	Liver	MRI / CT	−18.7	−1.6
2011	Collins	Retrospective	30	9	800	−12.5	Liver	CT	−18	−2.0
2011	Edholm	Prospective	15	4	800–1100	−6.1	Liver	MRI	−12	−3.0
2004	Fris	Prospective	40	2	456	−4.2	Left lobe	Ultrasound	−5.1	−2.6
2013	Gonzalez-Perez	Prospective	20	6	800	−12.2	Liver	CT	−20.3	−3.4
2006	Lewis	Prospective	18	6	450–800	−9.1	Liver	MRI	−14.7	−2.5

### Meta-Regression Analysis

Taking the number of patients in each study as a weighing factor, the mean reduction in liver volume is −13.6 % (SD 5.8), which is the result of a mean number of 729 kcal per day (SD 286) during a mean number of 6.4 weeks (SD 3.6). Using the same weighing factor in a linear regression analysis, the resulting constant (β0) is −3.294, and the regression coefficients for the number of kilocalories and number of weeks (β1 and β2) are −0.002 and −1.372, respectively. In other words, a low calorie diet of 500 kcal during 3 weeks for example would result in a reduction in liver volume of (−3.294 + (500* −0.002) + (3*−1.372) = −8.41 %.

### Alternative Preoperative Measures

Three studies [12, 14, 21] investigated the effect of alternative preoperative measures on liver volume. Aberle et al. [12] studied the effect of 15 mg sibutramine for 6 weeks in a retrospective case-matched-control study. Patients in the group treated with sibutramine lost an average of 4.8 kg and the matched control group gained an average of 7 kg in 6 weeks. The greatest decrease was seen in the longitudinal measurements of the right hepatic lobe in the group treated with sibutramine (mean decrease of 1.9 ± 2.2 cm). Brody et al. [14] investigated the effect of Nuvista® together with a low calorie diet (1200–1500 kcal/day) for 4 weeks. Ianelli et al. [21] investigated the effect of 1500 mg omega-3 fatty acids a day for 4 weeks without any dietary restrictions. An overview of preoperative alternative measures is shown in Table 2.

**Table 2:** Overview of alternative preoperative measures

Year	Investigator	Study type	Patients (n)	Duration (weeks)	Type	Methods		Change in liver volume (%)	Change in liver volume per week (%)
						Liver or left lobe	Imaging technique		
2009	Aberle	Retrospective, case matched control	40 (20 vs. 20)	6	Sibutramine	Left lobe	Ultrasound	n.a.	n.a.
2011	Brody	Prospective	21	4	Nuvista® and low calorie diet	Left lobe	Ultrasound	−43.4	−10.9
2013	Ianelli	Prospective	20	4	Omega-3 fatty acids	Left lobe	Ultrasound	−20.0	−5.0

### Endoscopic Measures

Frutos et al. [19] investigated the effect of an intragastric balloon for 6 months prior to LRYGBP in super obese (BMI > 50 kg/m^2^). Mean BMI decreased from 55.2 ± 6.9 to 47.4 ± 7.7 kg/m^2^. Liver volume was measured by CT and decreased from 2938.5 ± 853.1 to 1918 ± 499.8 cm^3^ (31.8 %).

### Approach of Gastroesophageal Junction

Three studies [13, 15, 22] investigated the effect of a preoperative low calorie diet and reported a relatively easy approach of the gastroesophageal junction during LRYGBP. Colles et al. [15] also reported that none of the surgical procedures were converted from laparoscopic to open. Lewis et al. [22] also reported a soft liver that could easily be manipulated during surgery by using a very low calorie diet. Ianelli et al. [21] reported no difficulties in the approach of the gastroesophageal junction in patients treated with omega-3 polyunsaturated fatty acids. In addition, the liver could be easily retracted during surgery.

## Discussion

This study included 7 studies that investigated the effect of low calorie diets on liver volume in 6 different countries, including Israel, Australia, New Zealand, USA, Mexico, and Sweden. The duration and the content of the diets varied widely between the studies. All studies reported a reduction in liver volume varying from 5 to 20 %.

The lowest reduction in liver volume was associated with the shortest duration of the diet. Studies with a longer duration of the diet showed on average more reduction in liver volume by approximately 2.4 % per week. This indicates that the duration of the diet correlates with the amount of reduction of liver volume. However, the highest reduction in liver volume was not associated with the longest duration of the diet or the lowest amount of calories, suggesting that the content of the diet, i.e. the amount of protein, carbohydrates, and fat, also plays an important role in reducing liver volume.

Meta-regression analysis showed that the effect of the amount of calories is marginal compared to the effect of the number of weeks of the LCD. Although the number of studies is considered not sufficient for a robust meta-regression analysis, it is remarkable that the amount of calories has no effect on liver volume. These results suggest that any kind of diet will reduce liver volume and that the focus should lie on the duration of the diet and the adherence.

A downside of a low calorie diet is that it is relatively expensive and burdensome for the patient. Common side effects of dietary foods are nausea, vomiting, stomach discomfort, and constipation. In addition, patients often have difficulties with adherence to the diet. Another serious drawback of this method is that low calorie diets for multiple weeks may induce a catabolic state, which could be a disadvantage in recovery after surgery.

All studies that investigated the effect of low calorie diets had several limitations. First, all studies used a relative small number of patients, varying between 14 and 40 patients. Second, there was a lack of well-designed randomized studies. Six studies were prospective observational, 1 study was retrospective, only 1 study used a control group, and none of the studies was designed as a randomized controlled trial. In addition, none of the studies performed a power calculation.

Three studies investigated the effect of alternative measures to reduce liver volume. Aberle et al. [12] investigated the effect of sibutramine on BMI and liver volume compared to a case-matched-control group in a retrospective study. Sibutramine is a centrally acting serotonin-norepinephrine reuptake inhibitor developed for the treatment of obesity. It increases the metabolism; it induces an earlier feeling of satiety and is thereby a successful method to reduce weight. [23] Patients were advised to use sibutramine prior to surgery and retrospectively compared the group that had used sibutramine with a historic control group. This design has such a high probability of selection bias, that it is hard to draw any conclusions.

Brody et al. [14] studied the effect of the nutritional supplement Nuvista® combined with a low calorie diet. Nuvista® is a nutritional supplement that consists of a high protein content and a wide range of vitamins and minerals. They reported the greatest decrease in liver volume (43 %) of all studies. Unfortunately, they did not use a control group; so, the additional effect of Nuvista® cannot be determined. But, the combination of Nuvista® and a low calorie diets seems to be the most effect way to reduce liver size.

Ianelli et al. [21] investigated the effect of omega-3 fatty acids on liver volume and found a reduction of 20 % of the left hepatic lobe. The results of this study are promising, because they proved that it could be an effective method to reduce liver volume without the need for dietary restrictions. A limitation of this study is that they only reported the BMI at baseline and not after treatment with omega-3 fatty acids. Therefore, it is uncertain whether the reduction in liver volume was caused by the use of omega-3 fatty acids alone or that weight loss could also have played a role.

Only one study [19] reported on the effect of an intragastric balloon in order to reduce liver volume in super obese patients prior to LRYGBP. Downsides of this method are high risk of side effects and the invasive character of this method. Therefore, this method should only be reserved for super obese patients that require a considerable reduction of weight and liver volume in order to facilitate LRYGBP.

Three studies [13,15,22] that investigated the effect of a low calorie diet on liver volume reported that there were no problems with the approach of the gastroesophageal junction during surgery. In addition, pretreatment with omega-3 fatty acids also resulted in an easy approach of the gastroesophageal junction and in a soft liver that could easily be manipulated [21]. Since these two factors are the most important rationale behind preoperative treatment with a low calorie diet, it is remarkable that only four studies reported information on this matter.

Six studies [12, 14–17, 21] reported information about postoperative outcome. In general, there were no differences in the effect of preoperative methods on postoperative outcome. However, this review included not enough studies to investigate the effect of preoperative methods to reduce liver volume on postoperative outcome. In the most recent review on this topic by Cassie et al. [24], the authors concluded that there is not enough evidence to support the positive effects of preoperative diets on postoperative outcome.

Our study had several limitations. First, only a small number of studies with a relative small number of patients were included. Second, it was difficult to make a proper comparison of the studies because of their differences in study design. Third, there were different radiographic methods used to evaluate liver volume. In addition, some studies measured whole liver volume and some only the left hepatic lobe.

In our opinion, the best radiographic method to measure liver size and fat content is magnetic resonance imaging (MRI), because it gives an accurate estimation of liver size and fat content. Computed tomography (CT) gives an accurate estimation of liver size; however, liver CT is not the best method to measure steatosis [25]. At this moment, ultrasound (US) is most used to diagnose steatosis or steatohepatitis [26]. However, US is not the best method to measure liver size, especially not in morbidly obese subjects.

In conclusion, this systematic review showed that pharmacological measures and an intragastric balloon are more effective than low calorie diets in reducing liver volume prior to gastric bypass surgery. However, low calorie diet is the preferable method to reduce liver volume, considering the level of evidence and practical applicability. On average, low calorie diets reduce liver size by 2.4 % per week; therefore, we would recommend a low calorie diet of at least 4 weeks prior to surgery. If patients do not tolerate a low calorie diet, alternatives such as Nuvista® or omega-3 fatty acids should be considered. In the super obese, a gastric balloon is an effective method to prepare these patients for LRYGBP. There is a need for well-designed randomized studies with sufficient power in order to confirm the effectiveness of preoperative methods to reduce liver volume.
